# Cellular Structures Analysis Under Compression Test

**DOI:** 10.3390/polym17111476

**Published:** 2025-05-26

**Authors:** Maria C. Bedoya, J. William Restrepo, Luis V. Wilches, Johnnatan Rodriguez

**Affiliations:** Ingeniería Mecánica, Universidad EIA, Envigado 055428, Colombia; maria.bedoya83@eia.edu.co (M.C.B.); jose.restrepo15@eia.edu.co (J.W.R.); luis.wilches@eia.edu.co (L.V.W.)

**Keywords:** cellular structures, additive manufacturing, fuse filament fabrication

## Abstract

Cellular structures, formed by periodic two- or three-dimensional cells, offer weight reduction without compromising mechanical performance and are commonly fabricated via additive manufacturing. This study investigates the compressive behaviour of three polymer lattice structures—gyroid, diamond, and octet truss—fabricated by fused filament fabrication (FFF). A Box–Behnken experimental design was used to systematically evaluate the influence of three key parameters: cell size, strut/wall thickness, and layer thickness. A total of 225 samples were produced using PLA and subjected to compression testing in accordance with ASTM D1621. Linear regression and response surface methodology were employed to determine the statistical significance and impact of each factor. The results indicate that cell size has the strongest influence on both maximum force and displacement, followed by strut/wall thickness and layer thickness. Among the configurations, gyroid structures had the highest strength-to-density ratio, while diamond structures had the highest deformation capacity. These findings provide design insights for optimising lattice structures in lightweight applications and highlight the importance of carefully balancing geometric and printing parameters in FFF-based polymer components.

## 1. Introduction

Cellular structures (CS) broadly refer to engineered materials that consist of periodic or aperiodic arrangements of interconnected elements—such as struts, walls, or surfaces—designed to achieve tailored mechanical or functional properties. Among them, lattice structures (LS) represent a particularly important subclass, characterized by repeating cellular patterns formed by interconnected struts and nodes. These architectures aim to minimize material usage and overall weight while maintaining mechanical performance [1,2]. LS have traditionally been limited to simple shapes such as foams and honeycombs due to the limitations of conventional manufacturing processes. However, the advent of additive manufacturing (AM) allows the production of geometries previously considered unattainable by traditional methods [3,4,5,6]. This technology now allows the fabrication of complex cellular designs suitable for a wide range of engineering applications [7,8,9,10,11].

LS exhibit desirable mechanical properties, including energy absorption capacity and high strength-to-weight ratio. These properties are highly valued in various fields of engineering. For instance, in biomedical applications, LS are suitable for implants that mimic bone porosity and improve osseointegration compared to solid prosthetics [7,11,12]. In aerospace, LS facilitate the design of structurally efficient, high-performance components [11,13,14]. In addition, LS have significant potential in thermal management systems, where their increased surface area contributes to improved heat transfer efficiency [8].

The mechanical performance of CS strongly depends on the morphology, which is typically classified into three main categories: triply periodic minimal surfaces (TPMS), trusses, and planar structures [4,15]. Some of these morphologies are modelled after crystalline lattice configurations—based on scaffold-like frameworks, planar transformations, or minimal surface geometries—while others are inspired by natural or biological forms. In general, the geometry of CS is determined by three primary parameters: cell size and the thickness or diameter of the struts or walls. These variables largely determine the mechanical behaviour of the structures, depending on their morphological classification [5,16,17].

Recent advances in additive manufacturing have enabled the exploration of increasingly complex polymer lattice structures for lightweight and energy-absorbing applications. However, the majority of research has focused either on metallic lattices or on isolated geometric variables, with limited integration of process parameters relevant to polymer-based systems. Studies have shown that the compressive behaviour of these structures is highly dependent on both geometry and fabrication conditions, but few works have systematically evaluated their interaction [18,19].

Several additive manufacturing techniques have been shown to be suitable for producing lattice structures with varying degrees of complexity and material requirements. For metallic lattices, powder bed fusion (PBF) and electron beam melting (EBM) are among the most widely used methods, offering high resolution and structural integrity [5,6,10,17,18]. These processes enable the production of intricate geometries in metals such as titanium alloys and stainless steels, which are commonly used in biomedical and aerospace components [7,11,17]. For polymer lattices, resin photopolymerization and fused filament fabrication (FFF) are commonly used, providing design flexibility and accessibility for prototyping and low-load functional parts [1,2,3,16,20,21,22,23].

FFF is widely used in industry for rapid prototyping, a process that enables the rapid fabrication of physical models or components directly from digital designs [16,21,22,23,24,25,26,27]. This approach is often used in the early stages of product development to evaluate form, fit, and function prior to full-scale production. However, the capabilities of FFF extend beyond prototyping, enabling the production of end-use components with increasing reliability. Key parameters that affect the FFF process include layer thickness, print temperature, print speed, infill pattern and infill density [21,27,28]. Additional settings such as retraction and de-retraction (which control the reversal and resumption of filament extrusion to minimize stringing and defects) can also be optimized during 3D printing. This technique, based on a layer-by-layer manufacturing approach, is widely used in rapid prototyping and other industrial and functional applications [21,22,23,24,25,26,27,28]. However, the influence of these secondary settings on the result remains comparatively small.

Previous studies have investigated the relationship between printing parameters, cell geometry, and their combined influence on mechanical performance, but this relationship needs further investigation. Using commercial filaments, layer thickness has been identified as the most influential printing parameter, while other settings are generally less significant than geometric variables [27,28]. This study addresses the need for a structured experimental framework to evaluate the combined effects of geometry and process settings in FFF lattice structures. The present study focuses on three key factors, cell size, layer thickness, and strut or wall thickness (or diameter), and evaluates their individual and combined influence on the compressive behaviour of three lattice morphologies fabricated by FFF. A Box–Behnken design was implemented to apply response surface methodology (RSM), providing a structured experimental framework that maps the interaction between geometric and process-related parameters.

## 2. Materials and Methods

**Figure 1:**
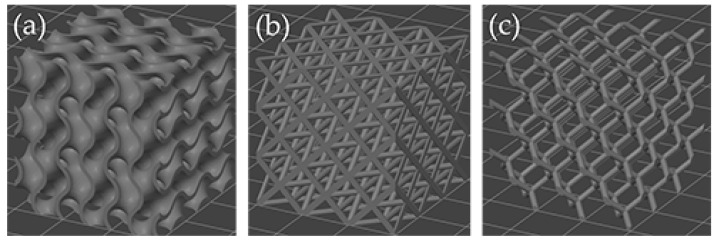
LS selected for the present study. (**a**) Gyroid, (**b**) octet truss, (**c**) diamond.

**Figure 2:**
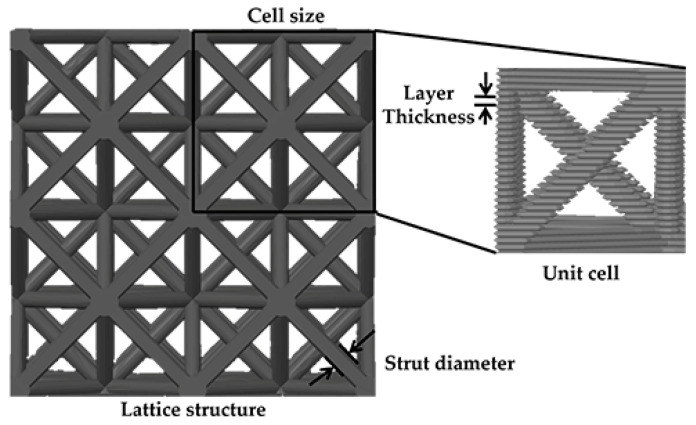
Key factors for LS analysis.

According to various characteristics previously identified in the literature review, three LS configurations were selected for analysis: gyroid, diamond, and octet truss (Figure 1). Figure 2 shows the CAD designs, a representation of the software slicing for printing, and the analysis of the LS focused on three key factors: cell size, layer thickness, and strut or wall thickness (or diameter).

The LS were modelled using nTopology software (5.0.4) [29] within a volume of 30 × 30 × 30 mm. Fabrication was performed by 3D printing using a Prusa MK4 printer (Prusa, Prague, Czech Republic) [30], without an enclosed chamber, at the Universidad EIA. Printing parameters were defined using PrusaSlicer software (2.8.0) [31]. The material used was a polylactic acid (PLA) filament, brand 4DLab (Dosquebradas, Colombia) [32]. A total of 225 LS samples were fabricated by combining three cell sizes (side lengths: 10 mm, 15 mm, and 20 mm), three strut/wall thicknesses (1 mm, 1.2 mm, and 1.4 mm), and three layer thicknesses (0.1 mm, 0.2 mm, and 0.3 mm). To evaluate the mechanical properties of the LS, compression tests were performed on an Instron 3345 machine (resolution +/− 0.5%, maximum load 5KN) at the Universidad EIA (Instron, Norwood, MA, USA). Compression tests were performed in accordance with ASTM D1621 [33], producing stress-strain curves and measurements of maximum force and displacement at failure.

The resulting data were processed and analysed using response surface methodology. Each response was evaluated individually to characterize the mechanical behaviour of the LS. A second-order Box–Behnken design [34,35,36,37,38] was used to define the experimental matrix, with three factors at three levels: cell size, strut/wall thickness, and layer thickness. The experimental conditions are summarised in Table 1.

## 3. Results and Discussion

**Figure 3:**
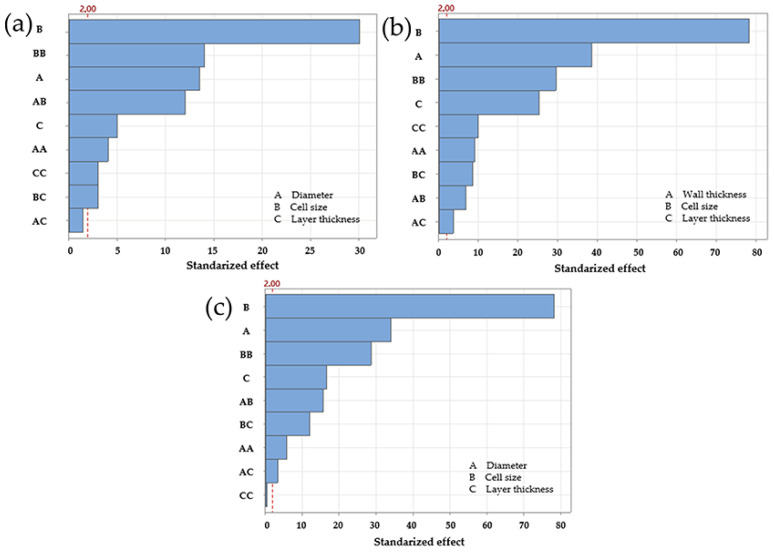
Regression and sensitivity analysis for maximum force in LS. (**a**) Diamond, (**b**) gyroid, (**c**) octet truss.

Once the compression tests were completed under the parameters defined by the experimental design, a linear regression analysis was carried out to assess the influence of each factor on the maximum force. This statistical approach not only determines the significance of individual parameters such as cell size, strut diameter, and layer thickness but also allows their interactions to be assessed. The *p*-value is used as a criterion of statistical significance; values below 0.05 indicate that a parameter or interaction has a significant effect on the mechanical response. Table 2 shows the *p*-values obtained for each parameter and their interactions, summing the results for each lattice structure. While Table 2 shows which factors are statistically significant, Figure 3 illustrates the relative influence of each factor, highlighting their contribution to the overall mechanical behaviour.

Linear regression for the diamond LS, based on maximum force, yielded an R^2^ of 95.91% and a predicted R^2^ of 94.33%, indicating a good fit between the model and the experimental data. The Pareto chart identified cell size as the most influential factor. This was followed by the second order term for cell size, strut diameter, the interaction between diameter and cell size, and layer thickness. Regression coefficients supported these findings, as shown in Figure 3a. However, *p*-value analysis indicated that the interaction between diameter and cell size was not statistically significant, as its *p*-value exceeded 0.05. It also confirmed the lack of significance for the interactions between diameter and layer thickness and between cell size and layer thickness, both with *p* values greater than 0.05 (Table 2).

For the gyroid LS, the regression results showed an R^2^ of 99.31% and a predicted R^2^ of 99.05%, suggesting a better fit than that observed for the diamond configuration. The Pareto chart highlighted cell size as the dominant factor, followed by strut/wall thickness, the second order term for cell size, and layer thickness. The *p*-values for all interactions remained below 0.05, confirming their significance and supporting the trends observed in the Pareto chart (Figure 3b and Table 2). On the other hand, the regression analysis for the octet truss LS produced an R^2^ of 99.26% and a predicted R^2^ of 98.99%, placing its accuracy between that of the diamond and gyroid models. On the other hand, the regression analysis for the octet truss LS produced an R^2^ of 99.26% and a predicted R^2^ of 98.99%, placing its accuracy between that of the diamond and gyroid models. Similarly, the Pareto chart identified cell size as the most influential factor, followed by strut diameter, the second-order term for cell size, and layer thickness. These results were consistent with both the regression coefficients and the *p*-value analysis. The only non-significant interaction was the second-order term for layer thickness, as indicated in both the Pareto chart and the statistical output (Figure 3c and Table 2).

Overall, the results confirm that cell size had the greatest influence on maximum force for all three lattice structures. The four most significant factors for the gyroid LS and octet truss LS were consistent, suggesting similar mechanical behaviour. In contrast, the interaction between cell size and strut diameter only ranked in the top four in the diamond configuration. It ranked fifth in the octet truss LS and second to last in the gyroid LS. This suggests that the relevance of this interaction was less than originally expected.

**Figure 4:**
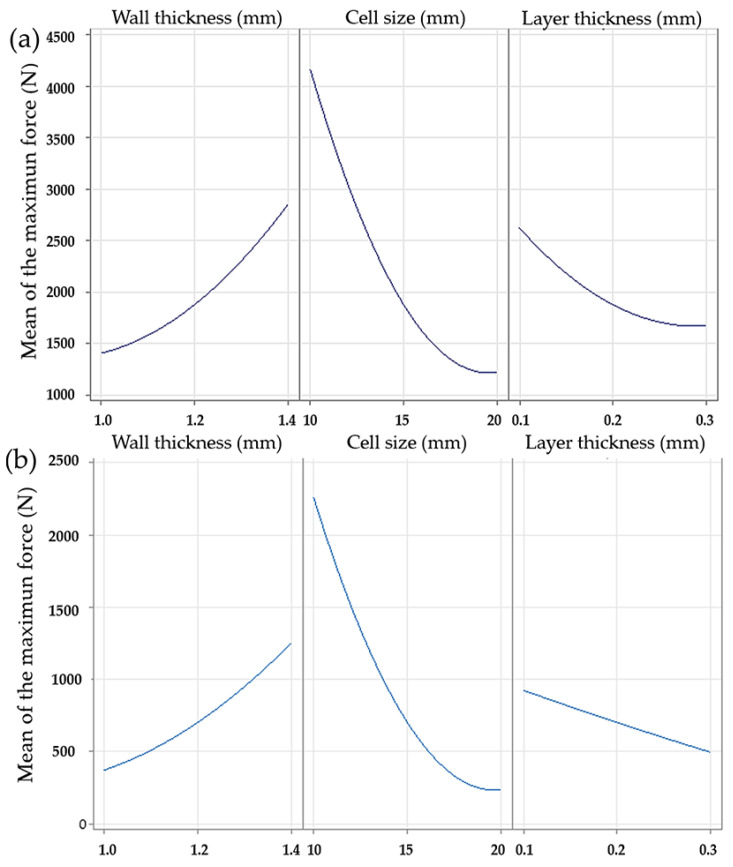
Main effects plots for maximum force. (**a**) Gyroid LS, (**b**) octet trust LS.

The main effects plots for the gyroid and octet truss LS showed similar trends in response to changes in strut diameter and cell size, as shown in Figure 4. In both cases, increasing strut diameter resulted in higher maximum forces, with values nearly doubling from 1 mm to 1.4 mm. The effect of cell size followed a quadratic pattern. A sharp decrease in maximum force was observed between 10 mm and 15 mm, while the difference between 15 mm and 20 mm was less pronounced. The effect of layer thickness differed between the two structures. In the gyroid LS, it followed a quadratic trend, while in the octet truss LS, it appeared linear. Despite this difference, thinner layers consistently resulted in higher maximum forces.

**Figure 5:**
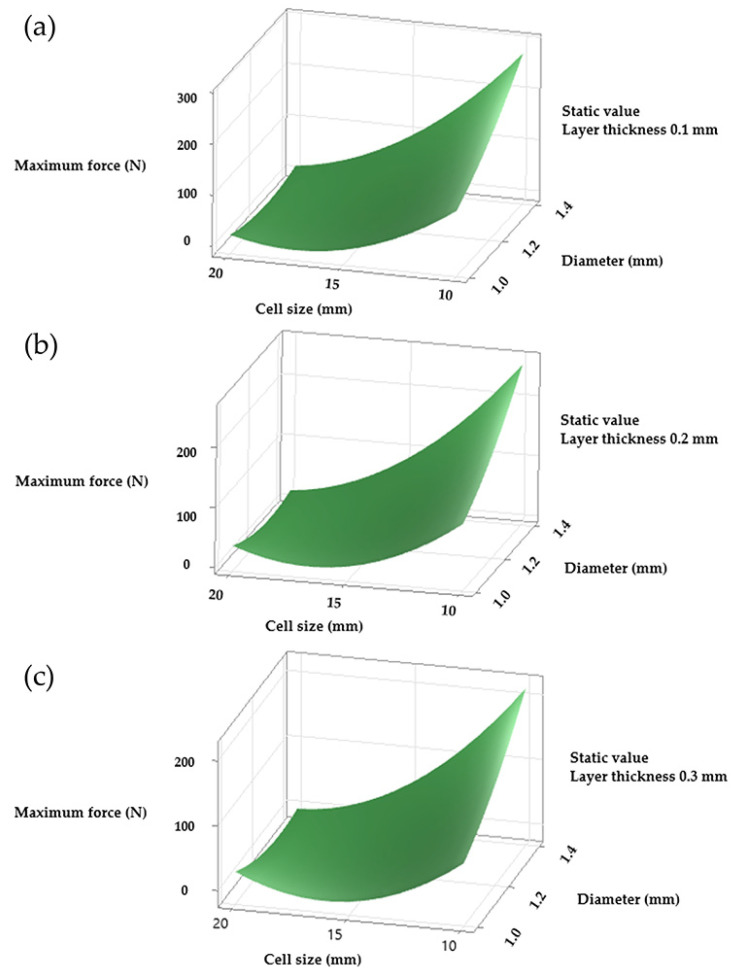
Main effects plots for maximum force for diamond structure. (**a**) With constant layer thickness of 0.1 mm, (**b**) 0.2 mm, (**c**) 0.3 mm.

For the diamond LS, the main effect plots were not available, which limited the direct comparison. However, response surface plots provided further insight into the combined effects of strut diameter and cell size. For all three structures, the configuration that produced the highest maximum force corresponded to a cell size of 10 mm and a strut diameter of 1.4 mm. Layer thickness also affected the response in the gyroid and octet truss LS, although its effect was more pronounced in the diamond configuration (Figure 5).

To evaluate the effect of the experimental design parameters on the maximum displacement of the lattice structures, a linear regression analysis was conducted. As in the case of maximum force, the goal was to verify the statistical significance of each factor and their interactions on the displacement response. Table 3 illustrates the *p*-values obtained for each parameter and their interactions, summarizing the results for each lattice structure.

**Figure 6:**
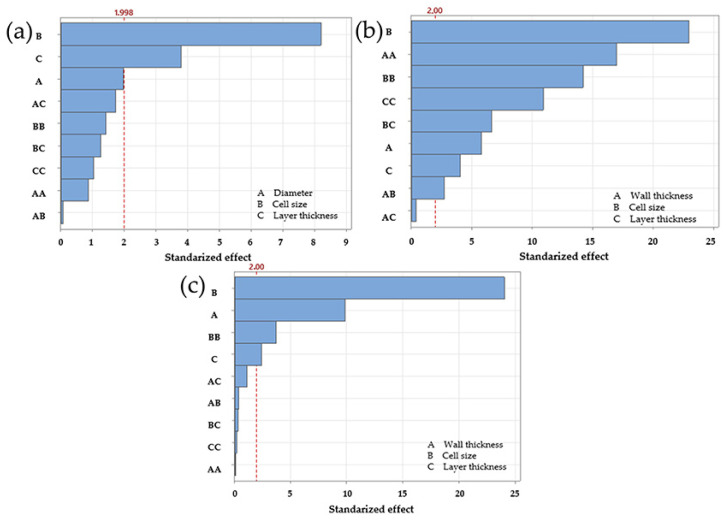
Linear regression results for the maximum displacement. (**a**) Diamond, (**b**) gyroid, (**c**) octet truss.

The accuracy of the linear regression models for maximum displacement decreased significantly for all three structures, with the diamond configuration showing the weakest fit. It yielded an R^2^ of 60.09% and a predicted R^2^ of 44.7%, indicating poor agreement between the experimental data and the model. The Pareto chart identified cell size and layer thickness as the only relevant factors. However, none of the interactions showed statistical significance as confirmed by *p*-value analysis (Figure 6a, Table 3). Attempts to model only linear effects further reduced the R^2^, suggesting potential inconsistencies in the displacement data for the diamond LS. The gyroid LS achieved the best model fit with an R^2^ of 94.69% and a predicted R^2^ of 93.42%. The Pareto chart and corresponding *p*-values indicated that all interactions were significant except for the one between strut diameter and layer thickness (Figure 6b, Table 3). For the octet truss LS, the regression model yielded an R^2^ of 91.44% and a predicted R^2^ of 88.79%. The associated Pareto chart identified cell size as the most influential factor, followed by strut diameter, the second-order term for cell size, and layer thickness. The remaining interactions were not statistically significant, as confirmed by both the graph and the *p*-value analysis (Figure 6c, Table 3).

**Figure 7:**
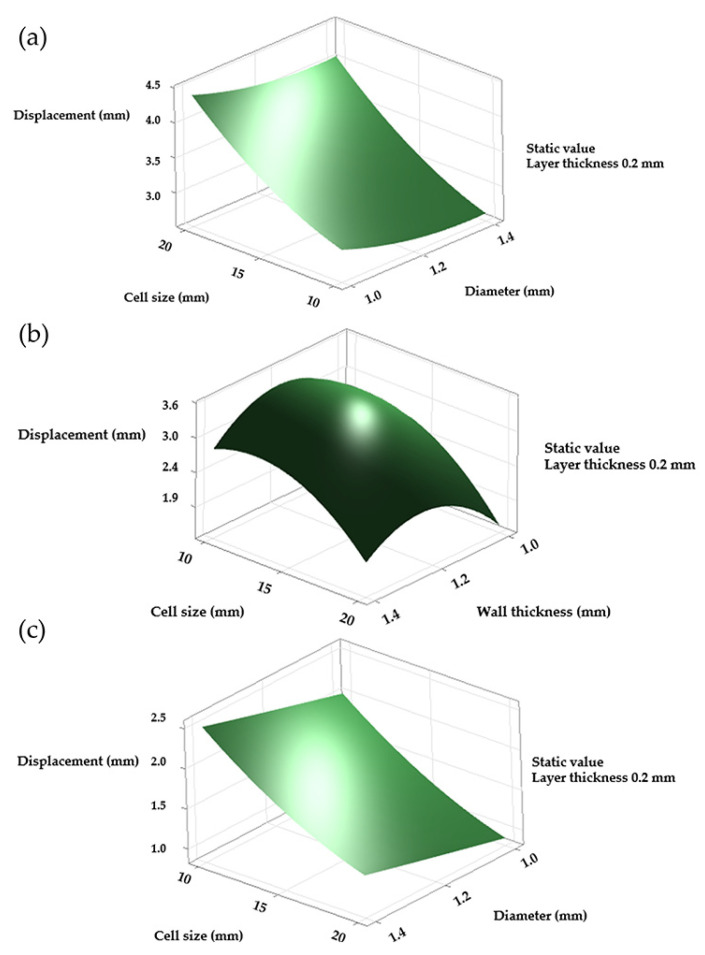
Main effects plots for maximum displacement at a layer thickness of 0.2 mm. (**a**) Diamond, (**b**) gyroid, (**c**) octet truss.

Although the results for the diamond LS were considered unreliable due to its low R^2^ value, the response surfaces indicated that maximum displacement occurred when the cell size reached 20 mm and the strut diameter was 1 mm. A thinner layer also appeared to increase displacement. In the gyroid LS, maximum displacement was observed with a cell size of 10 mm and a strut diameter of 1.2 mm. However, variations in strut thickness produced minimal differences. In contrast, layer thickness had a more pronounced effect, with larger values associated with increased displacement. The octet truss LS showed a similar trend to the gyroid in terms of displacement behaviour (Figure 7).

From the regression analysis based on maximum force, cell size emerged as the most influential factor in all three structures, followed by strut/wall thickness (diameter) and layer thickness. The influence of layer thickness was more pronounced in the gyroid and octet truss configurations than in the diamond LS. This result is consistent with the idea that for a given total volume, smaller cell sizes provide a greater surface area for stress distribution [16,21,22,38,39]. Consequently, the structure exhibits greater resistance to compressive loading. Cell size therefore plays a central role in determining load capacity. In comparison, the influence of strut diameter is less significant in shaping the overall geometry. Film thickness affected the resolution and precision of the printed solids but had a more modest effect on mechanical strength. In terms of maximum displacement, cell size again emerged as the dominant factor. For the gyroid and octet truss LS, interactions involving strut diameter and layer thickness also contributed significantly to the response. This effect was not seen in the diamond LS. These results suggest that cell size was the primary parameter determining mechanical performance in terms of both maximum force and displacement.

**Figure 8:**
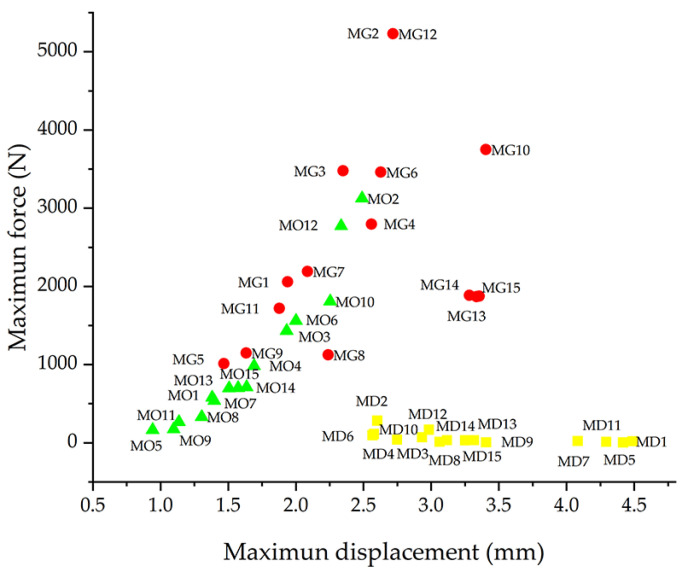
Comparison of maximum force and displacement across LS. The nomenclature indicates the LS morphology: MD stands for diamond (yellow circles), MG stands for gyroid (red circles), and MO stands for octet truss (green triangles).

The performance of the three structures was further evaluated using Wilcoxon nonparametric tests. This method was chosen because the data did not follow a normal distribution, and the samples were paired according to the experimental design. Pairwise comparisons were performed in the following order: Gyroid vs. diamond, gyroid vs. octet truss, and octet truss vs. diamond. The results indicated that the gyroid LS achieved the highest maximum force, followed by the octet truss LS, and then the diamond LS. In terms of maximum displacement, the diamond LS had the largest values, followed by the gyroid and the octet truss configurations. These comparisons are summarised in Figure 8, which shows the average of five replicates per sample and highlights the differences between the configurations.

The parameter configuration that optimised the maximum supported force for all three structures corresponded to a cell size of 10 mm, a strut diameter of 1.4 mm, and a layer thickness of 0.1 mm (Figure 5 and Figure 8; samples M2 and M12). The same combination also produced the greatest displacement in the gyroid and octet truss LS (Figure 7 and Figure 8, samples M2, M10, and M12), although the trend was not consistent in the diamond configuration. Further investigation of the diamond LS samples, especially those with results that deviate from the average behaviour, may be necessary. The force data, on the other hand, showed high reliability.

Diamond LS had greater displacements than the gyroid and octet truss configurations. On average, their values were nearly twice those of the octet truss LS and significantly higher than the gyroid LS. Samples with a 10 mm cell size showed less displacement than those with a 20 mm cell size, reflecting greater stiffness. Gyroid LS samples near the centre of the experimental design (Figure 8; MG13, MG14, MG15) recorded unexpectedly high displacements, suggesting that intermediate cell sizes may maintain elevated levels of deformation. In terms of maximum force, the gyroid LS outperformed both the octet truss and diamond configurations. Some octet truss LS samples matched the diamond LS at larger cell sizes, but as cell size decreased, their behaviour more closely matched that of the gyroid LS—demonstrating adaptable mechanical performance.

**Figure 9:**
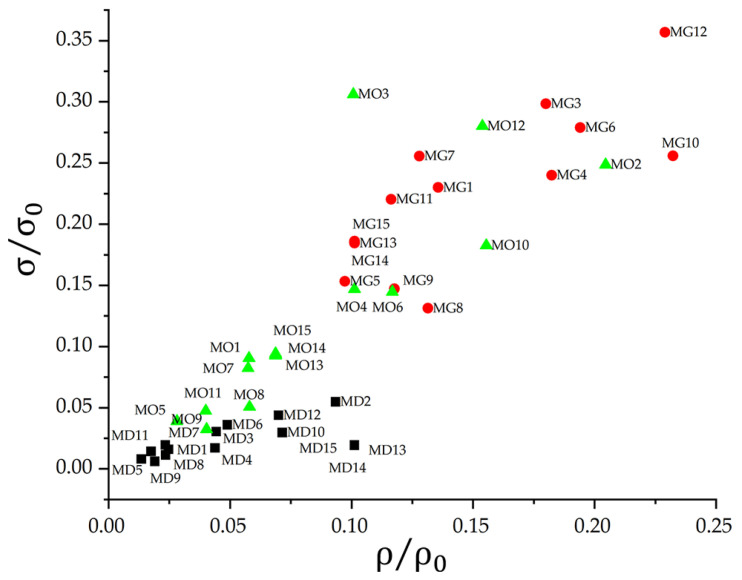
Comparison of normalised strength as a function of relative density across LS. The nomenclature indicates the LS morphology: MD stands for diamond (black rectangles), MG stands for gyroid (red circles), and MO stands for octet truss (green triangles).

The results support the selection of the gyroid LS for lightweight applications where high compressive strength and controlled displacement are required. The octet truss configurations may be better suited for structural components due to their rigidity. For biomedical applications that require ductile behaviour under low loads, diamond LS that retain their shape after failure remain promising. Following this analysis, stress values were estimated based on the average cross-sectional area along the height of each structure, taking into account geometric variation. The behaviour of the fifteen configurations is shown in Figure 9, which compares specific density with normalized stress.

All structures significantly reduced the effective density, with values below 25% of the original material. Despite the reduction in strength, the gyroid LS retained approximately 35% of its original strength at a relative density of 22%. This favourable result suggests that lower density gyroid LS designs could outperform the base material when normalized. Some octet truss LS samples exhibited a similar trend. In contrast, diamond LS maintained a nearly proportional relationship between strength and density, closely reflecting the behaviour of the bulk material. The gyroid LS thus combined low density with high strength. With the octet truss LS, performance varied more across configurations. Certain arrays achieved strength-to-density ratios comparable to or exceeding those of the gyroid LS. However, the mechanical response of the diamond was more variable. Their irregularity suggests that reducing cell size and increasing strut diameter may be necessary to improve both strength and stability.

**Figure 10:**
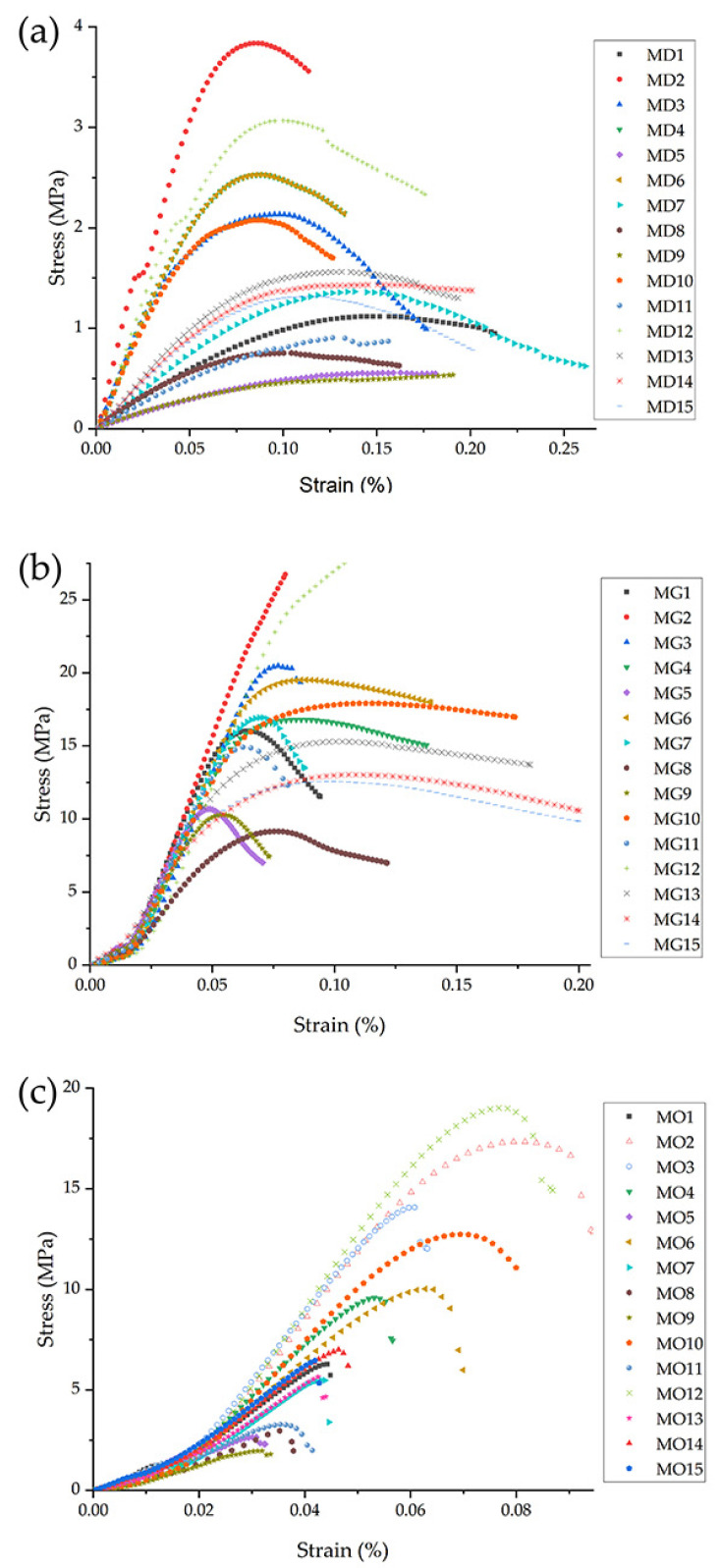
Stress–strain curves for representative configurations. (**a**) Diamond LS, (**b**) gyroid LS, (**c**) octet truss LS.

To better understand the structural behaviour, stress-strain curves were generated for each configuration. Figure 10 shows the mechanical response of the three morphologies across their 15 configurations. The gyroid LS exhibit a relatively consistent modulus of elasticity (E), while the diamond and octet truss lattice structures exhibit greater variability depending on their geometric configuration and dimensional printing parameters. The Young’s modulus of the diamond LS is particularly sensitive to these parameters, regardless of printing defects such as local variations in solidification rate, which cause some variation in compressive strength values. Reducing the cell size (from 20 mm to 10 mm) significantly increases E in the diamond and octet truss lattice structures by factors of 5 to 7 and 1.3 to 2.3, respectively. However, in the gyroid LS, reducing the cell size results in a reduction of E by a factor of 0.61 to 0.99. Similarly, reducing the print layer thickness while keeping the cell size and strut diameter constant increases E by a factor of 1.2 to 1.3 for the diamond LS and by a factor of 1.2 to 1.4 for the octet truss configuration. In contrast, for the gyroid LS, a reduction in film thickness results in a decrease in E by a factor of 0.7 to 0.9.

For all three structures, increasing the strut diameter (diamond and octet truss) or wall thickness (gyroid) results in an increase in E. Specifically, E increases by factors of 1.4 to 3.4 for the diamond LS, 1.2 to 1.93 for the octet truss LS, and 1.05 to 1.4 for the gyroid LS. The pronounced increase in stiffness for the diamond LS is likely due to its lower number of struts per node, which makes it more responsive to changes in strut diameter, layer thickness, and cell size. In this structure, the combination of increasing strut diameter and decreasing layer thickness improves energy dissipation by increasing the contact area between successive extruded layers. In the octet truss LS, the effects on E are less pronounced due to the higher number of struts, where symmetry distributes compressive loads more evenly. The gyroid LS, on the other hand, shows minimal sensitivity to pressure parameters due to its continuous and compact shape, where intersecting walls and curved surfaces efficiently absorb or dissipate compressive energy. Overall, the gyroid LS has the highest stiffness of the three configurations evaluated under compressive loads.

**Figure 11:**
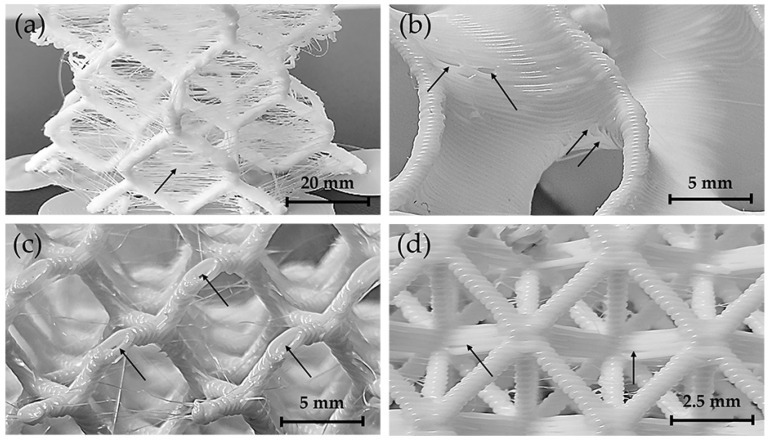
LS defects observed after 3D printing conformation. (**a**) Filament remnants, (**b**) printing discontinuities the gyroid LS, (**c**) strut variations, (**d**) printing discontinuities in the octet truss LS. Defects are are highlighted by arrows.

An optical inspection revealed localised imperfections, as shown in Figure 11, which are randomly distributed across the printed lattice structures. These defects are highlighted by the arrows. Thin polymer filaments or “stringing” were observed in the diamond LS (Figure 11a), caused by residual viscoplastic flow of the polymer as the nozzle moves between deposition points. These superficial imperfections do not affect the mechanical performance of the structures and can usually be eliminated by adjusting the printing parameters.

In contrast, the presence of voids between adjacent extrusion layers—observed in the gyroid LS (Figure 11b), diamond LS (Figure 11c), and octet truss (Figure 11d) LS—can affect the mechanical behaviour. The magnitude of this effect depends on both the extent of the voids along the polymer interface and the direction in which the compressive load is applied. In addition, the greater the density of these interfacial voids, the more likely they are to weaken the structure. These defects can be caused by abrupt changes in nozzle direction, causing local variations in polymer flow and cooling rate, resulting in incomplete bonding between layers. Although the print parameters were carefully optimised, localised overheating and nozzle contact occasionally caused struts to break during production. This problem was particularly severe in the diamond LS with 20 mm cell sizes and thinner struts, which had more surface irregularities than other morphologies. The presence of both interface discontinuities and defects in printed LS limits the ability to accurately model their mechanical behaviour, as these structures behave as morphologically and structurally discontinuous solids. Under compressive loads, these imperfections act as stress concentrators that induce fracture in the struts (diamond and octet truss) and walls (gyroid) of the printed components

**Figure 12:**
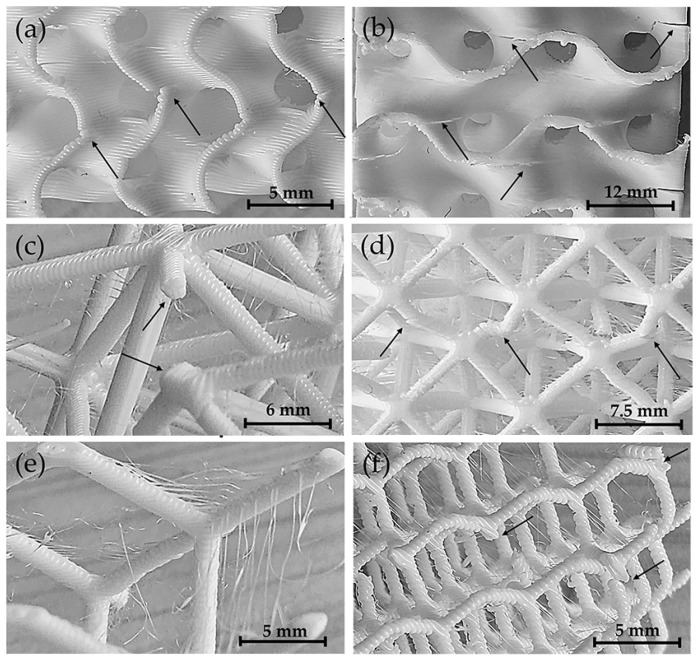
Failure modes observed in different configurations. (**a**) Gyroid LS M6, (**b**) gyroid LS M3, (**c**) octet truss LS M1, (**d**) octet truss LS M12, (**e**) diamond LS M1, (**f**) diamond LS M12. The arrows indicate the main points of failure in the structures.

Beyond their role as stress concentrators, the low density of these imperfections suggests a minor influence on overall mechanical performance. Instead, the structural response is primarily determined by the energy dissipated along the interfaces between the polymer layers. As the interface area increases, so does the ability of the structure to redistribute internal stresses during compression. Gyroid LS consist of continuous curved walls, creating a larger total surface area compared to the struts in diamond and octet truss LS. As a result, gyroid LS exhibits greater toughness and gradual failure behaviour (Figure 12a,b), attributed to progressive energy dissipation from the load application surface (top) to the supporting surface (bottom).

In octet truss and diamond LS, the interface area is smaller, and energy distribution is likely to occur through the joint nodes between the struts. As the number of struts decreases, the ability to distribute the load decreases, resulting in a more abrupt failure. For the octet truss LS, a reduced number of internal nodes resulted in sudden failure (Figure 12c), whereas a higher node density promoted a more gradual failure mode (Figure 12d). For the diamond LS, the reduction in cell size increased node density, which improved strength and stiffness and allowed progressive deformation of the struts to failure (Figure 12e,f). It is worth noting that in the octet truss LS, each node connects 12 struts, whereas in the diamond LS, each node connects only four struts. As a result of this reduced connectivity, the diamond LS collapses under lower compressive loads and exhibits greater ductility compared to octet truss structures (Figure 10).

## 4. Conclusions

This study evaluated the influence of geometric and printing parameters on the mechanical performance of three different lattice structures under compression loading. The findings highlight key factors affecting strength, deformation, and structural efficiency, providing valuable insights for optimizing lightweight cellular designs.

Cell size was the most influential factor for both maximum force and displacement in all three structures, as confirmed by the *p*-value analysis (*p* < 0.001 in all cases) and supported by the regression models. However, this dominance must be interpreted with caution, as the range of variation (10–20 mm) inherently produces greater differences in geometry and material distribution than the more subtle changes in strut diameter (1–1.4 mm) or layer thickness (0.1–0.3 mm).

The gyroid LS exhibited the highest average compressive strength, with peak force values more than 5000 N in samples with 10 mm cell size and 1.4 mm wall thickness. This structure also showed the most consistent response across the design space, with an R^2^ value of 99.31% and a predicted R^2^ of 99.05% in the force model.

The diamond LS, on the other hand, showed the largest displacements, averaging up to 6.5 mm under compression. However, it also had the lowest R^2^ (60.09%) in the displacement model, suggesting higher experimental variability or sensitivity to small geometric changes.

The octet truss LS showed intermediate mechanical behaviour, with good compressive strength and moderate displacement, making it a robust option where balanced performance is required.

The most favourable configuration in all cases was a 10 mm cell size, 1.4 mm strut/wall thickness, and 0.1 mm layer thickness, which maximised compressive strength and provided adequate ductility in both the gyroid and octet truss lattice structures. While all lattice structures contributed to a significant reduction in relative density (below 25% of solid material), the gyroid LS achieved the best strength-to-density ratio, confirming its suitability for lightweight applications where both stiffness and energy absorption are required.

These findings provide design guidance for optimising the mechanical performance of polymer lattice structures, highlighting the importance of carefully balancing geometric parameters and print resolution. Future work should include fatigue testing and validation under different loading modes to extend the applicability of the proposed configurations.

## Figures and Tables

**Table 1:** Box–Behnken design defined tests.

Nomenclature	Strut/Wall Thickness (mm)	Cell Size (mm)	Layer Thickness (mm)
M1	1.4	20	0.2
M2	1.4	10	0.2
M3	1.4	15	0.1
M4	1.4	15	0.3
M5	1	20	0.2
M6	1	10	0.2
M7	1	15	0.1
M8	1	15	0.3
M9	1.2	20	0.3
M10	1.2	10	0.3
M11	1.2	20	0.1
M12	1.2	10	0.1
M13	1.2	15	0.2
M14	1.2	15	0.2
M15	1.2	15	0.2

Note: The number indicates the experimental configuration. The nomenclature then includes an intermediate letter indicating the LS morphology—D for diamond (MD), G for gyroid (MG), and O for octet truss (MO).

**Table 2:** *p*-value for each force regression.

Parameters	*p* Values		
	Gyroid	Diamond	Octet
Diameter	0.000	0.000	0.000
Side	0.000	0.000	0.000
Thickness	0.000	0.033	0.000
Diameter × Diameter	0.000	0.435	0.000
Side × side	0.000	0.004	0.000
Thickness × thickness	0.000	0.577	0.664
Diameter × side	0.000	0.269	0.000
Diameter × thickness	0.001	0.946	0.002
Side × thickness	0.000	0.332	0.000

**Table 3:** *p*-value for each displacement regression.

Parameters	*p* Values		
	Gyroid	Diamond	Octet
Diameter	0.000	0.451	0.000
Side	0.000	0.125	0.000
Thickness	0.000	0.302	0.019
Diameter × Diameter	0.000	0.172	0.920
Side × side	0.000	0.619	0.000
Thickness × thickness	0.000	0.188	0.864
Diameter × side	0.008	0.803	0.713
Diameter × thickness	0.679	0.610	0.274
Side × thickness	0.000	0.351	0.751

## Data Availability

The original contributions presented in the study are included in the article, further inquiries can be directed to the corresponding author.
